# One-year longitudinal changes of peripheral CD4+ T-lymphocyte counts, gut microbiome, and plaque vulnerability after an acute coronary syndrome

**DOI:** 10.1016/j.ijcha.2024.101438

**Published:** 2024-06-04

**Authors:** Ana I Fernández-Avila, Enrique Gutiérrez-Ibanes, Irene Martín de Miguel, Ricardo Sanz-Ruiz, Álvaro Gabaldón, Francisco Fernández-Avilés, Josep Gómez-Lara, Marta Fernández-Castillo, Silvia Vázquez-Cuesta, Pablo Martínez-Legazpi, Nuria Lozano-Garcia, Elena Blázquez-López, Raquel Yotti, Igor López-Cade, Elena Reigadas, Patricia Muñoz, Jaime Elízaga, Rafael Correa, Javier Bermejo

**Affiliations:** a Department of Cardiology, Hospital General Universitario Gregorio Marañón, Facultad de Medicina, Universidad Complutense de Madrid, Instituto de Investigación Sanitaria Gregorio Marañón, and CIBERCV, Madrid, Spain; b Department of Cardiology, Hospital Universitario de Bellvitge, and CIBERCV, Barcelona, Spain; c Laboratory of Immune-Regulation, Instituto de Investigación Sanitaria Gregorio Marañón, Madrid, Spain; d Department of Clinical Microbiology and Infectious Diseases, Hospital General Universitario Gregorio Marañón, Facultad de Medicina, Universidad Complutense de Madrid, Instituto de Investigación Sanitaria Gregorio Marañón, and CIBERES, Madrid, Spain; e Department of Mathematical Physics and Fluids, Facultad de Ciencias, Universidad Nacional de Educación a Distancia, UNED, Madrid, Spain

**Keywords:** Acute coronary syndrome, Atherosclerosis, CD4+ T cells, Plaque vulnerability, Gut microbiome, Fibrotic cap thickness

## Abstract

**Background:**

Longitudinal changes in gut microbiome and inflammation may be involved in the evolution of atherosclerosis after an acute coronary syndrome (ACS). We aimed to characterize repeated profiles of gut microbiota and peripheral CD4+ T lymphocytes during the first year after an ACS, and to address their relationship with atherosclerotic plaque changes.

**Methods:**

Over one year we measured the microbiome, peripheral counts of CD4+ T populations and cytokines in 67 patients shortly after a first ACS. We compared baseline measurements to those of a matched population of 40 chronic patients. A subgroup of 20 ACS patients underwent repeated assessment of fibrous cap thickness (FCT) of a non-culprit lesion.

**Results:**

At admission, ACS patients showed gut dysbiosis compared with the chronic group, which was rapidly reduced and remained low at 1-year. Also, their Th1 and Th2 CD4+ T counts were increased but decreased over time. The CD4+ T counts were related to ongoing changes in gut microbiome. Unsupervised clustering of repeated CD4+ Th0, Th1, Th2, Th17 and Treg counts in ACS patients identified two different cell trajectory patterns, related to cytokines. The group of patients following a high-CD4+ T cell trajectory showed a one-year reduction in their FCT [net effect = -24.2 µm; p = 0.016].

**Conclusions:**

Patients suffering an ACS show altered profiles of microbiome and systemic inflammation that tend to mimic values of chronic patients after 1-year. However, in one-third of patients, this inflammatory state remains particularly dysregulated. This persistent inflammation is likely related to plaque vulnerability as evident by fibrous cap thinning (Clinical Trial NCT03434483).

## 1 Introduction

Impaired local immune regulation in the arterial wall plays a major role in the pathogenesis of acute coronary syndromes (ACS). Particular populations of activated CD4+ T helper (Th) lymphocytes are involved in the rupture of atheroma plaques [1]. Because their increased presence has been consistently demonstrated in atherosclerotic lesions,[2] most research has focused on Th1 and Th17 subpopulations of CD4+ T lymphocytes. Th1 cells produce large amounts of tumor necrosis factor (TNF) and interferon gamma (IFN-γ), which impact plaque stability [3]. Th17 cells co-express IL-17 and IFN-γ and by promoting the apoptosis and death of smooth muscle cells in the fibrous cap, they increase plaque vulnerability [4]. In advanced stages of atherosclerosis, Th2-driven processes may contribute to plaque rupture [5]. Conversely, T regulatory (Treg) cells attempt to counterbalance these Th-mediated effects, increasing the plaque content of smooth muscle cells and collagen [6].

The involvement of CD4+ T populations in ACSs is less clear at a systemic level. Systemic inflammation is a well-known risk factor for ACSs, and systemic anti-inflammatory drugs lower the risk of ACSs [7]. In the early phases of an ACS, increased levels of circulating cytokines and activated CD4+ T helper cells [1], [8] may impair local regulation because circulating activated cells are attracted towards atherosclerotic lesions by plaque cytokines and chemokines [1].

These systemic immune cell populations are kept in a bidirectional homeostatic balance with gut microbiome. Furthermore, microbiota dysbiosis may be involved in the activation of pro-inflammatory processes causing plaque activation, rupture, and thrombosis [9]. Thus, over the time interplays between gut microbiome, systemic activation of CD4+ T lymphocytes, and plaque vulnerability may be involved in the pathogenesis of ACSs. This issue is particularly important after a first episode, because despite intensive secondary prevention, 12–15 % of patients with an ACS will suffer a recurrent ischemic event within the first year [10], [11]. Therefore, the present study was designed to investigate the longitudinal changes in gut microbiome and in CD4 + T cell counts during the first year after an ACS, and to address their relationship with atherosclerotic plaque vulnerability.

## 2 Methods

### 2.1 Study design and patient populations

**Fig. 1:**
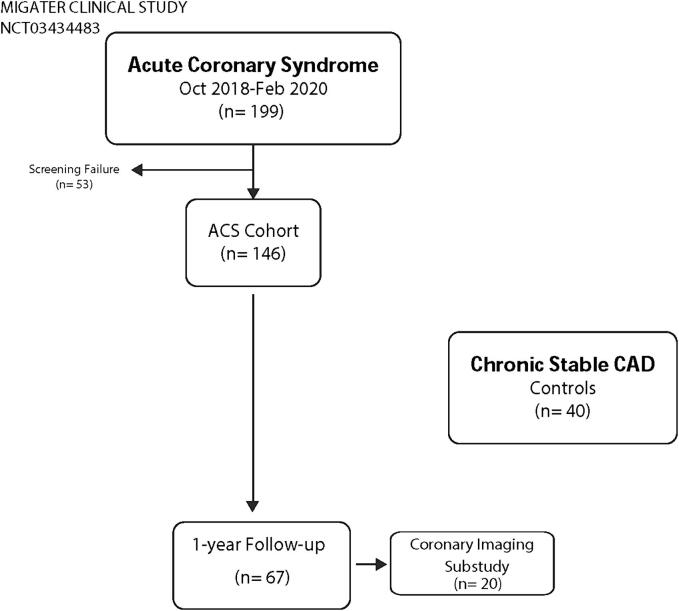
MIGATER Study Cohort. 67 ACS patients and 40 matched chronic controls are the basis of this report.

Adult admitted between October 2018 and February 2020 with a first established diagnosis of ACS were invited to enter the MIGATER observational longitudinal study (Fig. 1). Exclusion criteria are summarized in the Online Supplemental Table 1. At admission, and days 7, 30, 90, 180 and 365, patients underwent clinical revisions as well as stool and blood sampling. Following standard of care, Mediterranean diet was recommended to all patients. The 14-item dietary screener of Mediterranean diet (MEDAS)[12] was completed at inclusion and clinical visits. Data from repeated samples were available in 67 patients. For comparison, we selected a group of 40 age and sex matched patients with angiographically documented stable chronic coronary artery disease (CAD) following the following inclusion criteria: > 1-year free of cardiac events or admissions and following identical exclusion criteria than for the ACS group. In addition, a coronary imaging substudy was designed for ACS patients in whom an intermediate lesion could be angiographically identified in a non-culprit coronary vessel. In these patients, we performed an optical coherence tomography (OCT) examination of this intermediate lesion (<50 % stenosis) and repeated it at day 365.

The study protocol conforms to the ethical guidelines of the 1975 Declaration of Helsinki as reflected in the approval of the Ethics Committee of the Hospital General Universitario Gregorio Marañón, Madrid, Spain (CEIm 237/17) and was registered as NCT03434483. All patients signed informed consent for inclusion in the study.

### 2.2 Microbiota analysis

Total DNA was extracted from whole fecal samples. The hypervariable V4 region of the 16 s rRNA gene was analyzed by NGS (see Online Supplemental Material for details). We calculated alpha diversity using the Shannon index, which accounts for abundance and evenness of the species. Beta diversity, which captures changes in community composition, was calculated by the Bray-Curtis dissimilarity index in a non-msuetric multidimensional scaling (NMDS) using the phyloseq R package. Changes in microbiota abundance were analysed using ANCOMBC-2 [13].

### 2.3 Peripheral CD4 + T cell counts.

Fresh whole blood samples were employed for quantification of absolute counts (cells per μl) of CD4+ T cells. Absolute counts for Th0, Th1, Th2, Th17 and Treg populations were calculated by flow cytometry (Gallios cytometer, Beckman Coulter) using a combination of specific antibodies (Online Supplemental Figure 1) with flow-count fluorospheres (Beckman Coulter).[14].

### 2.4 Cytokine determination

We used a predesigned panel of 48 cytokines to measure in plasma samples using Olink Target 48 cytokine Plex (https://olink.com/products-services/target/48-cytokine-panel/) in a KTH-Scilifelab facility (Affinity Proteomics). We quantified C-reactive protein (CRP) from plasma samples using simple plex human CRP cartridge in an ELLA automated immunoassay system (Bio-Techne).

### 2.5 Coronary imaging

**Fig. 2:**
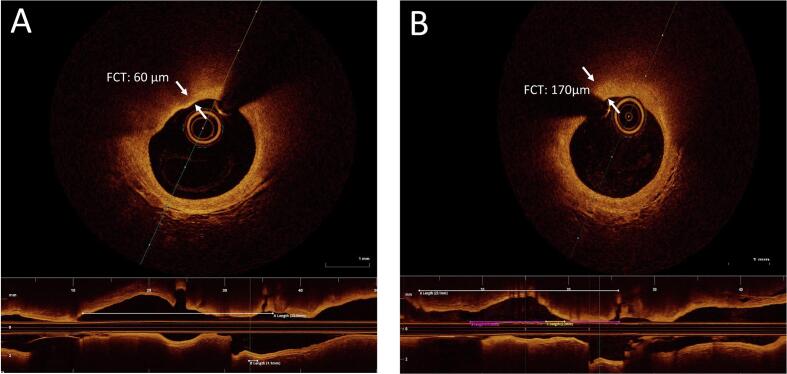
Change in Fibrous Cap Thickness. Baseline (A) and 1-year (B) evolution of fibrous cap thickness (FCT) measured by OCT in a non-culprit lesion in a patient with a low CD4+ T cell trajectory.

In those cases, we could angiographically identify an intermediate (<50 % stenosis) lesion in a non-culprit coronary vessel, we performed an automatic pullback of high-resolution OCT using a frequency-domain catheter (Dragonfly Optis, Abbott, MN, USA; see Online Supplemental Material for details). The same segment was re-imaged 1-year later during an elective catheterization procedure (Fig. 2). OCT analysis was blindly performed by an independent core-laboratory (BARCICORE).

### 2.6 Statistical analysis

Variables are expressed either as median [interquartile range] or estimated marginal means (95 % confidence interval), as indicated. All statistical models were adjusted for age, sex, and body mass index (BMI) as well as for the first six scores resulting from a multiple correspondence analysis integrating all cardiovascular risk factors and relevant ongoing medications at each visit (Online Supplemental Figure 2). We used multivariate analysis of variance as the global test to compare differences in cell populations and microbiota in ACS vs chronic controls, followed by one-way ANOVAs. Longitudinal changes were analyzed using mixed effects models (see Online Supplemental Material for details).

We performed an unsupervised multidimensional clustering of the longitudinal counts of the five CD4+ T cell subsets and cytokines using the *kml3d* algorithm for joint trajectories (see Online Supplemental Material for details). Baseline predictors of a high vs. low CD4+ T cell trajectory were identified using logistic regression and the c-index. Finally, we used a generalized estimating equation model to address the impact of a high vs. low CD4+ T cell trajectory on FCT and laboratory determinations. All statistical analyses were performed in R (v. 4.1.3); p-values < 0.05 and fold-changes in microbiome abundance > 1.5 were considered significant.

## 3 Results

### 3.1 Study population and clinical outcomes

ACSs were classified as unstable angina in 13 patients, non-ST elevation myocardial infarction (NSTEMI) in 22 and STEMI in 32. ACS and chronic patients showed no relevant clinical differences at entry (Online Supplemental Table 2) with a mean age of 60 [54 – 68] years old, 83 % were males, and BMI was 28 [26], [27], [28], [29], [30], [31] Kg/m^2^. There were significant differences in medications between ACS at entry and chronic controls that became comparable thereafter (Online Supplemental Table 3). A near-significant trend towards increasing Mediterranean diet adherence across time was observed in ACS patients (MEDAS mean score at inclusion: 8.8 ± 1.6; at 12-months: 9.8 ± 1.6; p = 0.05. Online Supplemental Figure 3). There were no major cardiac events during the 1-year follow-up period, except for one patient requiring repeated revascularization 15 days after admission in the target non culprit lesion.

### 3.2 Gut microbiome analyses

**Fig. 3:**
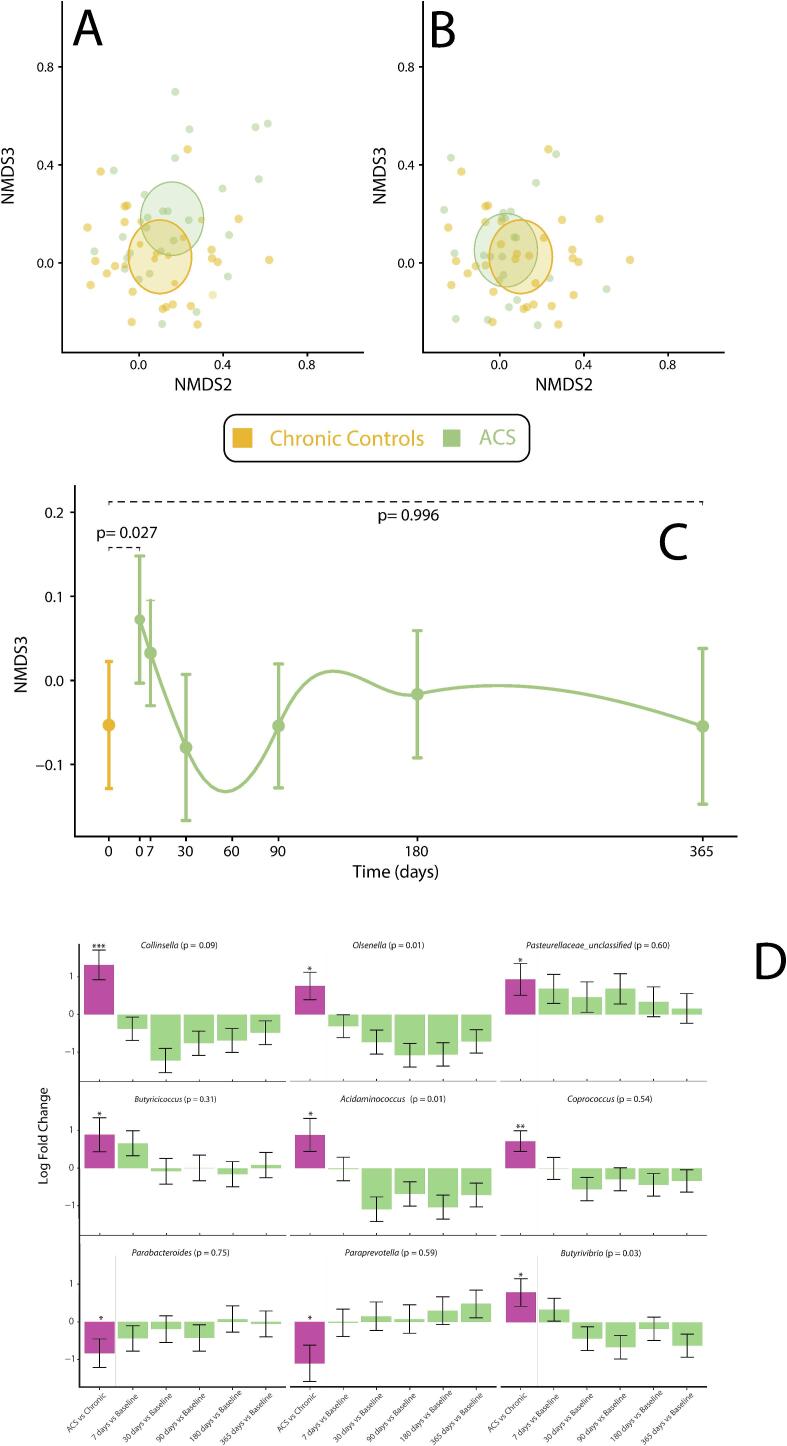
Beta Gut Microbiota Diversity.A: Bray-Curtis dissimilarity in non-metric multidimensional scaling in ACS at admission vs chronic controls. B: Bray-Curtis dissimilarity in non-metric multidimensional scaling in ACS at 1-year vs chronic controls. C: ACS vs chronic controls and longitudinal changes in NMDS3 Bray-Curtis dissimilarity. D: Trends in abundance, log fold changes, of bacteria genera in ACS patients. Significant ACS vs Chronic control differences: *: p < 0.05; **p < 0.005, ***p < 0.0005.

Values of alpha diversity were similar between chronic controls and ACS patients at admission. However, beta-diversity of ACS patients at inclusion was significantly different from chronic controls (p = 0.005), specifically the NMDS3 Bray-Curtis dimension (Fig. 3A–C); at 1-year, these differences disappeared (Fig. 3B–C).

The main difference in bacteria composition between ACS and chronic controls was a 2.5-fold positive change of *Collinsella* genus in the former (p < 0.001; q = 0.08; Fig. 3D). Other Actinobacteria such as *Olsenella, Pasteurellaceae* and *Butyrivibrio*, and *Butyricicoccus, Acidominococcus* and *Coprococcus* were also enriched in ACS patients compared to controls (fold-change > +1.5, p < 0.05). The opposite was detected for Paraprevotella and Parabacteroides (fold-change < -1.5, p < 0.05, Fig. 3D). In ACS patients, the enrichment of *Collinsella* rapidly decreased following a 2.3-fold change at 30 days (p < 0.001) and remaining low at 1 year (Fig. 3D). Similar significant trends were observed for *Olsenella*, *Butyrivibrio*, and *Acidominococcus* genera (Fig. 3D).

### 3.3 Th and Treg CD4+ Lymphocyte analyses

**Fig. 4:**
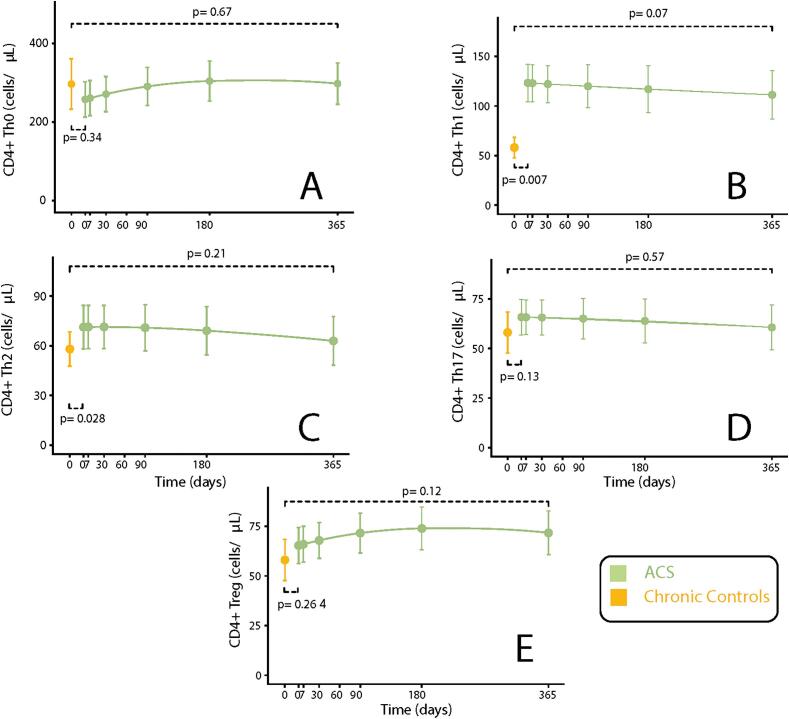
CD4+ T cell measurements in ACS vs Chronic Controls and Longitudinal changes. A: Th0 cell counts. B: Th1 cell counts. C: Th2 cell counts. D: Th17 cell counts. E: Treg cell counts.

Th and Treg cell counts were significantly different between in ACS patients at entry and the control group (Fig. 4). Specifically, counts of Th1 cells were higher in the ACS group than in controls (127 ± 9 vs. 90 ± 10 cells/µL, p = 0.007; Fig. 4B). However, Th1 cell counts gradually decreased in ACS subjects during follow-up (Fig. 4B). A similar behavior was observed for Th2 cells, with an initial overrepresentation in ACS patients compared to controls (73 ± 7 cells vs. 51 ± 8 cells/µL, p = 0.028) that gradually decreased towards control values at 1 year (Fig. 4C). The levels of Th17 cells followed a similar trend but neither differences from controls nor longitudinal changes were statistically significant (Fig. 4D). Also, counts for Th0 and Treg were not significantly different from controls and followed non-significant longitudinal changes (Fig. 4A and E).

**Fig. 5:**
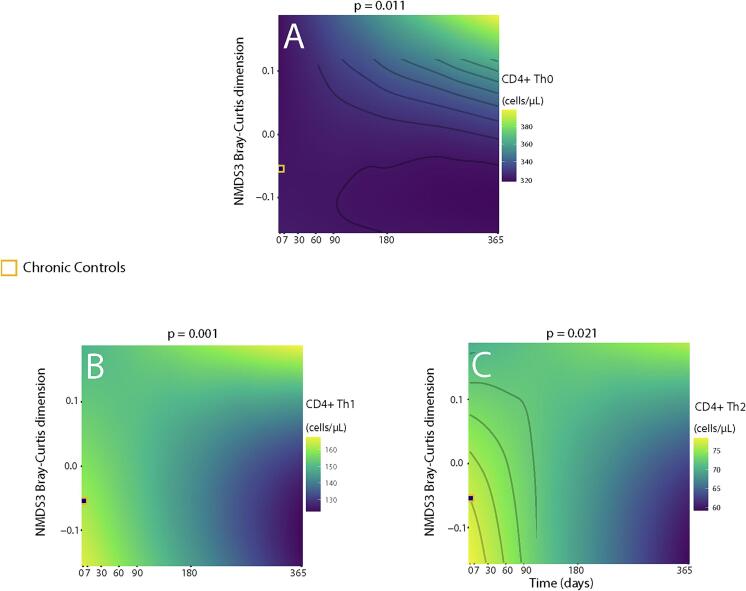
Interaction of Microbiota Diversity with CD4+ T Cell Counts in ACS patients Along Time. A: Th0 counts with NMDS3 Bray-Curtis dimension. B: Th1 counts with NMDS3 Bray-Curtis dimension. C: Th2 counts with NMDS3 Bray-Curtis dimension.

**Fig. 6:**
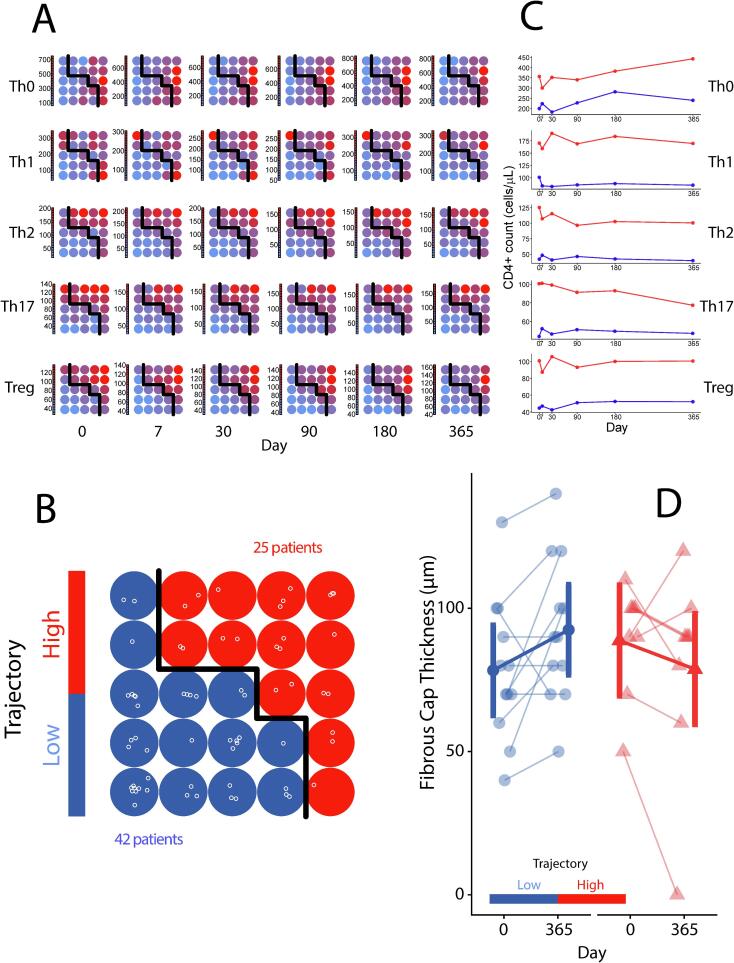
Identification of CD4+ T cell trajectories and Their Relationship with the Fibrous cap thickness. Unsupervised clustering of longitudinal CD4+ T cell counts. A: SOMs of ACS patients clustering by CD4+ T cell counts along time. B: Longitudinal CD4+ T cell cluster trajectories. C: Summary of SOMs per patient profile. D: Association analysis with atheroma fibrotic cap thickness.

Significant longitudinal interactions were observed between Th cell counts and microbiome diversity in ACS patients (Fig. 5). Compared to values from chronic controls, ACS patients with impaired beta-diversity (NMDS3) values from 180 days on, showed higher Th0, Th1 and Th2 cell counts until the end of the year (Fig. 5). Most importantly, the individual longitudinal changes of the CD4+ T cell populations followed two distinct patterns identified by unsupervised multidimensional clustering of the repeated measures of Th0, Th2, Th1, Th17, and Treg (Fig. 6A): trajectories were clustered as high and low cell counts in 25 (37 %) and 42 subjects (63 %), respectively, as clearly shown in the self-organizing map (SOM) (Fig. 6B). Patients showing high baseline cell counts followed higher parallel trajectories for all five cell types throughout the full year and persisted higher at the end of the study (Fig. 6C). Patients showing a high cell-count trajectory were younger and with a higher BMI than those following a low count trajectory (Table 1). By multivariate logistic regression, the odds ratios (OR) for a high-count trajectory were 2.7 (95 % CI: 1.6 to 4.4) per +5 Kg/m^2^ of BMI and 0.7 (95 % CI: 0.6 to 0.8) per +5 years of age. Neither the type of ACS, nor any particular cardiovascular risk factor was associated with the cell count trajectory pattern. The c-index_boot_ for predicting a high vs. low cell trajectory based on BMI and age was = 0.78 (95 % CI: 0.68 to 0.86), increasing up to 0.97 (0.93 to 0.99) when Treg counts at admission were included in the model.

**Table 1:** Baseline Clinical Characteristics of patients with ACS clustered in High and Low CD4+ T Cell Counts.

**Variable**	**Overall**, N = 67	**High cell count**, N = 25	**Low cell count**, N = 42	**p-value**
Age	60 [54–68]	55 [48–62]	62 [55–70]	0.004
Sex				>0.999
Female	10 (15 %)	4 (16 %)	6 (14 %)	
Male	57 (85 %)	21 (84 %)	36 (86 %)	
Body Mass Index (Kg/m^2^)	29 [26–31]	31.1 [27.7–33.4]	27.1 [25.6–30.4]	0.001
Hypertension	31 (46 %)	10 (40 %)	21 (50 %)	0.427
Dyslipidemia	35 (52 %)	12 (48 %)	23 (55 %)	0.592
Diabetes Mellitus				0.855
Type 1	1 (1.5 %)	0 (0 %)	1 (2.4 %)	
Type 2	16 (24 %)	7 (28 %)	9 (21 %)	
No	50 (75 %)	18 (72 %)	32 (76 %)	
Tobacco				0.120
Active Smoker	20 (30 %)	4 (16 %)	16 (38 %)	
Previous Smoker	23 (34 %)	9 (36 %)	14 (33 %)	
No	24 (36 %)	12 (48 %)	12 (29 %)	
Alcohol	6 (9.0 %)	4 (16 %)	2 (4.8 %)	0.186
Type of ACS				0.847
NSTEMI	13 (19 %)	4 (16 %)	9 (21 %)	
STEMI	32 (48 %)	13 (52 %)	19 (45 %)	
UA	22 (33 %)	8 (32 %)	14 (33 %)	

Values are shown either as (%) or median [IQR]. ACS: acute coronary syndrome; NSTEMI: non-ST segment elevation myocardial infarction; STEMI: ST segment elevation myocardial infarction; UA: unstable angina.

Similarly, the unsupervised clustering of CCL19, CCL2, CCL3, CRP, EGF, FLT3LG, IL6, OLR1, OSM and TNFSF12 repeated measures, also identified two high and low cytokine trajectories (Online Supplemental Figure 4). Although expressions of these cytokines fluctuated over time in the high trajectory, expressions in this trajectory were higher along the follow-up period. In fact, a high cytokine trajectory was significantly associated to the high CD4+ T cell trajectory (p < 0.001). There was no relationship between the high vs. low cell trajectory and the one-year changes in conventional biochemical, hematological, or coagulation determinations (Online Supplemental Table 4).

### 3.4 Fibrous cap thickness

The coronary imaging substudy demonstrated a significant association between the CD4+ T cell count trajectory and the one-year evolution of FCT of the non-culprit coronary lesion: patients following a high-count trajectory showed a reduction in FCT from [estimated marginal mean (95 % CI)] 88.8 (76.0 to 101.5) to 78.8 (55.4 to 102.0) µm, whereas patients following a low count trajectory showed an increase in FCT from 78.3 (65.1 to 91.6) to 92.5 (78.8 to 106.2) µm. The net effect of a low vs. high count trajectory was +24.2 (4.5 to 43.8) µm (p = 0.016; Fig. 6C). Intracoronary thrombus was identified attached to a plaque with no fibrous cap in a patient with a high cell-count trajectory. One-year changes in plaque volume and plaque burden were not associated to CD4 + T cell trajectories (Table 2).

**Table 2:** OCT measurements of the non-culprit coronary lesion in the coronary imaging substudy according to a High vs. Low CD4 + T cell trajectory.

**Variable**	**High Cell Count,** N = 8		**Low Cell Count,** N = 12	
	**Baseline**	**1-year**	**Baseline**	**1-year**
Vessel				
LAD	5 (62 %)		6 (50 %)	
RCA	1 (12 %)		4 (33 %)	
Circumflex	2 (25 %)		2 (17 %)	
Macrophage Grade				
1	0 (0 %)	1 (12 %)	2 (17 %)	3 (25 %)
2	1 (12 %)	2 (25 %)	1 (8.3 %)	4 (33 %)
3	6 (75 %)	5 (62 %)	7 (58 %)	4 (33 %)
4	1 (12 %)	0 (0 %)	2 (17 %)	1 (8.3 %)
Plaque Volume (mm^3^)	65 (41, 108)	65 (38, 110)	76 (38, 105)	67 (36, 121)
Plaque Burden (%)	64.2 (56.4, 68.0)	63.5 (59.0, 72.5)	62.5 (54.1, 65.4)	61.3 (52.1, 62.9)
Fibrous Cap Thickness (µm)*	95 (85, 100)	90 (75, 92)	75 (68, 92)	90 (78, 105)

Values show either n (%) or median (IQR). LAD: left anterior descending coronary artery; RCA: right coronary artery. *p < 0.05 for the baseline vs. 1-year changes high vs low cell trajectories analyzed using generalized estimating equation models.

## 4 Discussion

The present study describes the longitudinal changes that take place in microbiome and peripheral CD4+ T cell counts in the year following an ACS, and how the individual inflammatory response may impact the vulnerability of additional atherosclerotic plaques. T helper cells play a key proatherogenic role in ACS,[15] whereas Treg modulates the excessive activation.[16] Compared to chronic controls, in the present study we found increased counts of Th1, Th2, Th17 and Treg cells in patients with ACS. Importantly, for the first time, we report the 1-year evolution of these populations. Although all populations globally decreased towards control values, we observed two clear patterns of persistently low and high cell counts, along with a set of cytokines, readily recognizable at the time of admission.

Gut microbiome is believed to participate in the pathogenesis of atherosclerosis due to its impact on systemic inflammation. In recent cross-sectional studies, significant correlations were found between particular gut bacteria genera, microbial metabolites and quantitative plaque parameters.[17], [18] Furthermore, experimentally induced dysbiosis in mice suggests a role of acquired immunity activation as the intermediary link between dysbiosis and atherosclerosis development.[9] In our study, we report for the first time the 1-year evolution of gut microbiota and inflammation in ACS patients and we detected significant interactions between the time trajectory of individual T cell counts and gut microbiome composition. Shifts in gut microbial composition were highest in the relative abundance of *Collinsella*, a genera previously associated with symptomatic atherosclerosis in humans, but also widely associated to inflammation and obesity by altering gut permeability.[19], [20] In fact, low-calorie diets improves gut microbiota diversity and reduces the presence of *Collinsella*, particularly in obese type 2 diabetic subjects.[21] Likewise, the high Th1 cell counts characteristic of obesity, particularly in children and young adults,[22] support our observed risk factors for a persistent inflammation profile. Alterations in *Collinsella*, *Olsenella*, *Pasteurellaceae, Butyrivibrio, Butyricicoccus, Acidominococcus*, *Coprococcus*, *Paraprevotella* and *Parabacteroides* have been also detected in human atherosclerosis-related conditions in cross-sectional studies,[23] but, to our knowledge, the evolution of these microbiota alterations throughout the progression of the disease have never been reported before.

Fibrous cap thickness as assessed by OCT has been prospectively validated as a surrogate of plaque vulnerability and future events.[24] Although the evolution of FCT had never been linked to trajectories of systemic inflammation, several acute-phase observational studies anticipated this relationship.[25], [26] Reduced FCT in the culprit lesion is related to white cell counts,[27] monocyte/lymphocyte ratios,[28] as well as to the Th1 CD28^null^ cells [29] and pentraxin 3.[30] And most importantly, the beneficial effects of high dose statins[31], [32], [33] have been demonstrated to increase FCT. Remarkably, in the latter two trials the net effects of alirocumab [+29.6 (11.7 to 47.5) µm][33] and evolocumab [+21.2 (4.7 to 37.7) µm][32] were very close to the +24.2 (4.5 to 43.8) µm net difference we observed between cell trajectories.

Non-invasive biomarkers could be useful to identify patients at highest risk of ischemic events after an ACS. Our data suggest that patients who will follow a dysregulated inflammatory response, persistently elevated Th0, Th1, Th2, Th17 and Treg counts, during the first year can be adequately identified at admission based on age, BMI and CD4+ Treg cell counts. Previous results of patient profiling based on immune and inflammatory biomarkers support these findings.[34] If further confirmed, accurate identification of patients at risk could be useful to select candidates for systemic anti-inflammatory therapies.[7] Most healthy diets reduce cardiovascular risk.[35], [36], [37] These diet interventions impact inflammation through several pathways including a reduction of toxic microbiota metabolites.[20] Our results indicate that treatments targeting the interplay of diet, microbiota and immune modulation should be further explored. Large scale longitudinal population-based studies are needed to confirm whether the observed relationships play a substantive role in the pathogenesis of the ACS event.

## 5 Study limitations

The observational nature of our study precludes inferring causality. The study was underpowered to identify differences in clinical events and the coronary imaging substudy was performed on an even smaller subset of patients, therefore, large scale cohorts are needed to validate our results. The reported effects on plaque vulnerability are based on an intermediate non-culprit lesion in patients shortly after suffering and ACS, consequently associations with first coronary events cannot be directly established. Intensive cardiovascular drug therapies used after an ACS influence the immune response and gut microbiota composition.[38] Although we underwent comprehensive statistical adjustments, some of the observed differences could be due to these confounding factors. However, the relatively slow changes in microbiota diversity and CD4+ T cell populations, at a time when medications were fairly stable, suggest that this potential effect is negligible. Although a trend to increase Mediterranean diet adherence was observed during follow-up, because we did not systematically monitor diet adherence and did not implement diet biomarkers in our study, we cannot infer the role of diet modifications in our observed longitudinal behaviors.

## 6 Conclusions

Irrespective of the clinical presentation, compared to chronic CAD subjects, patients admitted with an ACS suffer of gut dysbiosis characterized by an enrichment in *Collinsella* genus among others. Intertwined to dysbiosis, ACS patients follow 1a systemic inflammatory state, characterized by elevated CD4+ Th1 and Th2 cell populations, and increased cytokine production, which Treg cells attempt to compensate. In roughly one-third of patients, this inflammatory state is particularly dysregulated and exaggerated inflammation will persist over 1-year. Inflammation with persistently high peripheral cell counts is likely associated with increased plaque vulnerability due to fibrous cap thinning of pre-existent atheroma of non-culprit lesions.

## CRediT authorship contribution statement

**Ana I Fernández-Avila:** . **Enrique Gutiérrez-Ibanes:** Writing – review & editing, Supervision, Methodology, Formal analysis, Conceptualization. **Irene Martín de Miguel:** Writing – review & editing, Investigation, Formal analysis, Data curation. **Ricardo Sanz-Ruiz:** Writing – review & editing, Methodology, Investigation, Data curation. **Álvaro Gabaldón:** Writing – review & editing, Methodology, Investigation, Data curation. **Francisco Fernández-Avilés:** Writing – review & editing, Supervision, Resources, Project administration, Funding acquisition, Conceptualization. **Josep Gómez-Lara:** Writing – review & editing, Writing – original draft, Resources, Methodology, Formal analysis. **Marta Fernández-Castillo:** Writing – review & editing, Investigation, Data curation. **Silvia Vázquez-Cuesta:** Writing – review & editing, Methodology, Investigation, Data curation. **Pablo Martínez-Legazpi:** Writing – review & editing, Visualization, Resources, Data curation. **Nuria Lozano-Garcia:** Methodology, Investigation, Data curation. **Elena Blázquez-López:** Writing – review & editing, Supervision, Methodology, Investigation. **Raquel Yotti:** Writing – review & editing, Conceptualization. **Igor López-Cade:** Writing – review & editing, Visualization, Methodology, Data curation. **Elena Reigadas:** Writing – review & editing, Supervision, Methodology, Investigation. **Patricia Muñoz:** Writing – review & editing, Supervision, Funding acquisition, Conceptualization. **Jaime Elízaga:** Writing – review & editing, Supervision, Resources, Funding acquisition. **Rafael Correa:** Writing – review & editing, Supervision, Methodology, Funding acquisition, Formal analysis, Conceptualization. **Javier Bermejo:** Writing – review & editing, Writing – original draft, Supervision, Resources, Methodology, Investigation, Funding acquisition, Formal analysis, Conceptualization.

## Declaration of competing interest

The authors declare that they have no known competing financial interests or personal relationships that could have appeared to influence the work reported in this paper.
