# Supplementary material for: One-year longitudinal changes of peripheral CD4+ T-lymphocyte counts, gut microbiome, and plaque vulnerability after an acute coronary syndrome
Source: Int J Cardiol Heart Vasc. 2024 Jun 4;53:101438. doi: 10.1016/j.ijcha.2024.101438 (PMC11190720; doi:10.1016/j.ijcha.2024.101438)
Supplement: Supplementary Data 1 [file mmc1.docx]

**One-year Longitudinal Changes of Peripheral CD4+ T-Lymphocyte Counts, Gut Microbiome, and Plaque Vulnerability After an Acute Coronary Syndrome**

**SUPPLEMENTARY MATERIAL**

Supplementary Material

[1 Supplementary methods 4](#_Toc166581642)

[1.1 detailed gut microbiota Analysis. 4](#_Toc166581643)

[1.2 Detailed coronary Imaging. 4](#_Toc166581644)

[1.3 detailed Statistical Analysis. 4](#_Toc166581645)

[2 Supplementary Tables 6](#_Toc166581646)

[2.1 Table 1. Exclusion criteria MIGATER cohort. 6](#_Toc166581647)

[2.2 Table 2. Baseline Clinical Characteristics. 7](#_Toc166581648)

[2.3 Table 3. Medication changes along the 1-year follow up in ACS patients. 8](#_Toc166581649)

[2.4 Table 4. Laboratory determinations of patients with ACS clustered in high and low cell counts. 9](#_Toc166581650)

[3 Supplementary Figures 10](#_Toc166581651)

[3.1 Figure 1. Representative flow cytometry plots for Th0, Th1, Th2, Th17 and Treg cell subset identification 10](#_Toc166581652)

[3.2 Figure 2. Variable contributions to cohort variability of the 6 main features of the dimensional reduction analysis for cardiovascular risk factors and medications. 11](#_Toc166581653)

[3.3 Figure 3. Adherence to the Mediterranean diet during follow-up assessed by 14-items MEDAS. 12](#_Toc166581654)

[3.4 Figure 4. Trends of the clustered cytokines significantly associated to high and low CD4+ T cell trajectories. 13](#_Toc166581655)

# Supplementary methods

## detailed gut microbiota Analysis.

Collected stool samples were stored at -80ºC until DNA isolation. Total DNA was extracted from 250 mg of the whole fecal samples using the QIAamp Fast DNA stool kit (QIAGEN). The hypervariable V4 region of the 16s rRNA gene was amplified by polymerase chain reaction (PCR) with 515-806 primers tailed with sequences to incorporate Illumina flow cell adapters and indexing barcodes. Sequencing was carried out on an Illumina Miseq platform (2 × 250). An average of 64,759 high-quality sequences (range 25,954 to 171,698) per sample were generated after merging and quality control. Sequenced data processing, operational taxonomic unit (OTU) clustering and taxonomic classification were performed using MOTHUR (v 1.39.5). Filtered sequences were aligned against the Silva reference database (v. 132). OTU counts were obtained by subsampling to the sample with the lowest sequences number. Finally, we classified OTU taxonomies using the RDP training set V.16 as reference. We calculated alpha diversity using the Shannon index, which accounts for abundance and evenness of the species present. Beta diversity, which captures changes in community composition, was calculated by the Bray-Curtis dissimilarity index in a non-metric multidimensional scaling (NMDS) using the *phyloseq* R package.

## Detailed coronary Imaging.

After angiographically identifying an intermediate (< 50% stenosis) lesion in a non-culprit coronary vessel, we performed an automatic pullback of high-resolution OCT using a frequency-domain catheter (Dragonfly Optis, Abbott, MN, USA). We administered contrast via a power injection to create a blood-free field, and recorded OCT images at 20 mm/s for a total of 54 mm from distal to proximal within the vessel. The first OCT examination was performed at the end of the clinical catheterization procedure after successful revascularization of the culprit lesion, within 48 hours of admission. The same segment was re-imaged 1-year later during an elective catheterization procedure. OCT analysis was performed by an independent core-laboratory (BARCICORE; Barcelona Cardiac Imaging core-laboratory) blinded to the clinical and blood test and microbiota data. Matched coronary segments between baseline and 1-year OCT images were selected according to clear anatomic landmarks. OCT quantitative analysis was performed at each 0.2 mm with the AptivueTM off-line software (Abbott, United States). Lumen area was automatically drawn by the software and manual corrections were performed if necessary. Vessel area was manually performed in all OCT cross-sections with visible external elastic membrane in >3/4 of the vessel perimeter according to previous publication. ^1^The most frequent plaque type (fibrous, fibrolipidic or fibrocalcific) and largest macrophage grade was assessed in matched study segments. ^1, 2^ In patients with fibrolipidic plaques, the minimal fibrous cap thickness (FCT) was measured at its thinnest point at baseline. Same measurements were performed in the matched cross-section at follow-up.

## detailed Statistical Analysis.

Variables are expressed either as median [interquartile range] or estimated marginal means (95% confidence interval), as indicated. All statistical models were adjusted for age, sex, and body mass index (BMI) as well as for the first six scores resulting from a multiple correspondence analysis integrating all cardiovascular risk factors and relevant ongoing medications at each visit (Supplemental Figure S1). We used multivariate analysis of variance (MANOVA) as the global test to compare differences in ACS vs chronic controls, followed by one-way ANOVAs. Microbiota abundance was analysed using ANCONBC-2.^3^ Longitudinal changes in CD4+ T cell counts were analyzed using mixed effects models entering time as a nonlinear fixed effect (in days after enrollment) using restricted cubic splines; the subject identifier was entered as the random effect. To address the relationship between microbiome and longitudinal changes in cell counts, we then repeated these models adding the interaction term of time with relevant diversity indices and plotted them using heatmaps.

We performed an unsupervised multidimensional clustering of the longitudinal counts of the five CD4+ T cell subsets studies using the kml3d algorithm for joint trajectories.^4^ Missing datapoints were first imputed incorporating both the information of available within-subject repeated measures and between-subject values at the missing timepoint, as recommended.^4^ The resultant number of clusters was automatically selected (n=2) based on the Chalinski and Harabatz criterion. Using this method, we reduced the repeated measurements of Th0, Th1, Th2, Th17, and Treg to two groups of high and low count trajectories, based exclusively on the cell-count data. The high and low trajectory clusters were plotted using self-organizing maps and individual trajectories. Cytokine trajectories were analyzed in an identical way than CD4+T cells. Baseline predictors of a high vs. low CD4+ T cell trajectory were identified using logistic regression and the c-index of the final model was calculated by bootstrapping of 500 replicates. Finally, we used a generalized estimating equation model to address the impact of a high vs. low CD4+ T cell trajectory on FCT and laboratory determinations. All statistical analyses were performed in R (v. 4.1.3); p-values < 0.05 and fold-changes in microbiome abundance > 1.5 were considered significant.

**References**

1. Gomez-Lara J, Oyarzabal L, Brugaletta S, et al. Coronary endothelial and microvascular function distal to polymer-free and endothelial cell-capturing drug-eluting stents. The randomized FUNCOMBO trial. *Rev Esp Cardiol (Engl Ed)*. 2021;74:1013-1022.

2. Tahara S, Morooka T, Wang Z, et al. Intravascular optical coherence tomography detection of atherosclerosis and inflammation in murine aorta. *Arterioscler Thromb Vasc Biol*. 2012;32:1150-7.

3. Lin H and Peddada SD. Analysis of compositions of microbiomes with bias correction. *Nat Commun*. 2020;11:3514.

4. Genolini C, Alacoque X, Sentenac M and Arnaud C. kml and kml3d: R packages to cluster longitudinal data. *J Stat Softw*. 2015;65:1 - 34.

# Supplementary Tables

## Table 1. Exclusion criteria MIGATER cohort.

|  |
| --- |
| **Exclusion criteria** |
| 1. Thrombolysis in Myocardial Infarction (TIMI) score <3 in the culprit vessel (unsuccessful revascularization) |
| 2. Killip classification III / IV |
| 3. Active systemic infection, chronic inflammatory disease, periodontal disease or treatment with corticosteroids, antibiotics or immunomodulators within the past 3 months |
| 4. Renal insufficiency with glomerular filtration rate less than 30 mL/min |
| 5. Severe hepatic insufficiency (liver cirrhosis in Child B or C stages). |

## Table 2. Baseline Clinical Characteristics.

| **Characteristic** | **ACS**, N = 67*^1^* | **Chronic**  **Controls**, N = 40*^1^* | **p-value***^2^* |
| --- | --- | --- | --- |
| Age | 60 [54 - 68] | 59 [54 - 67] | 0.652 |
| Sex |  |  | 0.497 |
| Female | 10 (15%) | 8 (20%) |  |
| Male | 57 (85%) | 32 (80%) |  |
| BMI | 29 [26 - 31] | 28 [26 - 30] | 0.803 |
| Hypertension | 31 (46%) | 25 (62%) | 0.104 |
| Dyslipidemia | 35 (52%) | 22 (55%) | 0.782 |
| Diabetes Mellitus |  |  | >0.999 |
| Yes | 17 (25%) | 10 (25%) |  |
| No | 50 (75%) | 30 (75%) |  |
| Tobacco |  |  | 0.932 |
| Ex-smoker | 23 (34%) | 14 (35%) |  |
| No | 20 (30%) | 13 (32%) |  |
| Active Smoker | 24 (36%) | 13 (32%) |  |
| Alcohol | 6 (9.0%) | 2 (5.0%) | 0.707 |
| ACS presentation |  |  |  |
| NSTEMI | 22 (33%) |  |  |
| STEMI | 32 (48%) |  |  |
| Unstable Angina | 13 (19%) |  |  |

^1^ n (%); Median [IQR] ^2^ Pearson's Chi-squared tests; Fisher's exact test; Wilcoxon rank sum test. ACS: Acute coronary syndrome; BMI: Body mass index; NSTEMI: Non ST-segment elevation myocardial infarction; STEMI: ST-segment elevation myocardial infarction.

## Table 3. Medication changes along the 1-year follow up in ACS patients.

| **Medication** | **Chronic CAD**,  N = 40*^1^* | **Baseline,**  N = 67*^1^* | **1 week,**  N = 67*^1^* | **1 month,**  N = 67*^1^* | **3 months,**  N = 67*^1^* | **6 months,**  N = 67*^1^* | **1 year,**  N = 67*^1^* |
| --- | --- | --- | --- | --- | --- | --- | --- |
| Diabetes medication |  |  |  |  |  |  |  |
| Insulin | 3 (7.5%) | 3 (4.5%) | 3 (4.5%) | 3 (4.5%) | 3 (4.5%) | 3 (4.5%) | 4 (6.0%) |
| Metformin | 4 (10%) | 14 (21%) | 16 (24%) | 16 (24%) | 16 (24%) | 16 (24%) | 16 (24%) |
| DPP4 Inhibitors | 2 (5.0%) | 5 (7.5%) | 5 (7.5%) | 4 (6.0%) | 4 (6.0%) | 4 (6.0%) | 4 (6.0%) |
| GLP1 agonist | 0 (0%) | 2 (3.0%) | 2 (3.0%) | 2 (3.0%) | 2 (3.0%) | 2 (3.0%) | 1 (1.5%) |
| SGLT2 Inhibitors | 2 (5.0%) | 2 (3.0%) | 3 (4.5%) | 3 (4.5%) | 2 (3.0%) | 2 (3.0%) | 4 (6.0%) |
| Proton pump inhibitors | 24 (60%) | 19 (28%) | 66 (99%) | 66 (99%) | 63 (94%) | 64 (96%) | 62 (93%) |
| Aspirin | 30 (75%) | 13 (19%) | 67 (100%) | 67 (100%) | 67 (100%) | 67 (100%) | 66 (99%) |
| Clopidogrel | 0 (0%) | 2 (3.0%) | 8 (12%) | 8 (12%) | 8 (12%) | 9 (13%) | 9 (13%) |
| Ticagrelor | 0 (0%) | 1 (1.5%) | 51 (76%) | 51 (76%) | 51 (76%) | 49 (73%) | 47 (70%) |
| Prasugrel | 0 (0%) | 0 (0%) | 8 (12%) | 8 (12%) | 8 (12%) | 8 (12%) | 8 (12%) |
| Acenocumarol | 5 (12%) | 1 (1.5%) | 2 (3.0%) | 2 (3.0%) | 2 (3.0%) | 1 (1.5%) | 2 (3.0%) |
| DOACs | 3 (7.5%) | 0 (0%) | 2 (3.0%) | 2 (3.0%) | 2 (3.0%) | 2 (3.0%) | 2 (3.0%) |
| Statins | 35 (88%) | 21 (31%) | 67 (100%) | 67 (100%) | 66 (99%) | 67 (100%) | 67 (100%) |
| Other lipid-lowering agents | 12 (30%) | 9 (13%) | 7 (10%) | 8 (12%) | 12 (18%) | 18 (27%) | 22 (33%) |
| Betablockers | 27 (68%) | 10 (15%) | 44 (66%) | 43 (64%) | 44 (66%) | 44 (66%) | 41 (61%) |
| ACEI/ARB | 32 (80%) | 25 (37%) | 57 (85%) | 55 (82%) | 55 (82%) | 54 (81%) | 54 (81%) |
| Antialdosteronics | 8 (20%) | 2 (3.0%) | 7 (10%) | 7 (10%) | 6 (9.0%) | 7 (10%) | 7 (10%) |
| Oral nitrates/ patch | 7 (18%) | 3 (4.5%) | 5 (7.5%) | 5 (7.5%) | 5 (7.5%) | 4 (6.0%) | 3 (4.5%) |
| Calcium antagonists | 15 (38%) | 9 (13%) | 7 (10%) | 8 (12%) | 9 (13%) | 9 (13%) | 8 (12%) |
| Furosemide | 7 (18%) | 3 (4.5%) | 1 (1.5%) | 1 (1.5%) | 2 (3.0%) | 2 (3.0%) | 3 (4.5%) |
| Other diuretics | 8 (20%) | 14 (21%) | 5 (7.5%) | 6 (9.0%) | 6 (9.0%) | 6 (9.0%) | 7 (10%) |
| Antidepressants | 7 (18%) | 7 (10%) | 4 (6.0%) | 4 (6.0%) | 4 (6.0%) | 7 (10%) | 6 (9.0%) |
| Anxiolytics  ^1^ n (%). DPP4: Dipeptidyl peptidase 4; GLP1: Glucagon-like peptide-1; SGLT2: Sodium-glucose co-transporter-2; DOACs: direct oral anticoagulants; ACEI: Angiotensin-converting enzyme inhibitor; ARB: Angiotensin receptor blocker. | 7 18%) | 4 (6.0%) | 1 (1.5%) | 1 (1.5%) | 4 (6.0%) | 3 (4.5%) | 3 (4.5%) |

## Table 4. Laboratory determinations of patients with ACS clustered in high and low cell counts.

^1^ Median [IQR]. LDH: Lactate dehydrogenase; CK: Creatine kinase; CPK: Creatine Phosphokinase; HDL: High-density lipoprotein; I.N.R.: International normalised ratio; APTT: Activated partial thromboplastin time; Nt-proBNP: N-terminal pro–B-type natriuretic peptide.

|  | **High cell count** N=22 | |  | **Low cell count** N=36 | |
| --- | --- | --- | --- | --- | --- |
| **Laboratory determinations** | **Baseline***^1^* | **1-year***^1^* |  | **Baseline***^1^* | **1-year***^1^* |
| ***Biochemistry*** |  |  |  |  |  |
| Calcium; mg/dL | 8.70 [3.83 - 4.22] | 9.10 [4.30-4.55] |  | 8.92 [3.80 - 4.20] | 9.00 [4.30 - 4.55] |
| Creatinine; mg/dL | 0.84 [0.76 - 0.94] | 0.79 [0.75 - 0.81] |  | 0.93 [0.76 - 1.03] | 0.88 [0.77 - 0.96] |
| Glucose; mg/dL | 115 [95 - 153] | 134 [108 - 150] |  | 102 [89 - 121] | 92 [83 - 101] |
| Potassium; mmol/L | 4.12 [3.89 - 4.40] | 4.60 [4.50 - 4.90] |  | 4.03 [3.70 - 4.26] | 4.20 [4.00 - 4.55] |
| Sodium; mmol/L | 139.00 [138.00 - 139.90] | 141.00 [139.00-143.00] |  | 140.00 [138.7-141.05] | 141.00 [140.00-142.50] |
| Proteins; g/dL | 6.63 [6.40 - 7.12] | 6.80 [6.30 - 7.00] |  | 6.40 [6.24 - 6.88] | 6.65 [6.32 - 6.90] |
| LDH; U/L | 215.00 [170.00 -362.50] | 146.00 [146.00 - 146.00] |  | 244.50 [191.75 - 373] | 195.00 [181.00 - 226.50] |
| Bilirubin; mg/dL | 0.56 [0.40 - 0.64] | 0.60 [0.40 - 1.00] |  | 0.80 [0.42 - 0.93] | 0.70 [0.50 - 0.95] |
| CK (CPK); U/L | 176.00 [103.50-253.50] | 181.00 [142.00-220.00] |  | 215.50 [116.25-334.75] | 111.00 [99.00-194.00] |
| Hemoglobin; g/dL | 15.00 [14.55- 15.85] | 14.60 [13.90- 15.60] |  | 14.00 [13.41- 14.88] | 14.00 [13.30- 15.35] |
| Nt-proBNP; ng/L | 297 [74 - 514] | 227 [93 - 374] |  | 465 [76 - 638] | 62 [59 - 96] |
| ***Lipid profile*** |  |  |  |  |  |
| Cholesterol; mg/dL | 188 [156 - 219] | 125 [111 - 152] |  | 152 [145 - 174] | 119 [101 - 138] |
| HDL cholesterol; mg/dL | 34 [32 - 39] | 46 [35 - 60] |  | 42 [35 - 49] | 45 [41 - 52] |
| Triglycerides; mg/dL | 140 [90 - 195] | 113 [92 - 124] |  | 119 [93 - 146] | 76 [62 - 108] |
| ***Coagulation*** |  |  |  |  |  |
| T. Prothrombin; seg. | 11.85 [10.90 - 12.83] | 11.40 [10.70 - 12.00] |  | 12.10 [11.80 - 12.90] | 11.50 [11.00 - 11.80] |
| I.N.R. | 1.02 [0.92 - 1.08] | 1.00 [0.99 - 1.04] |  | 1.03 [0.99 - 1.09] | 1.03 [1.00 - 1.07] |
| Fibrinogen; mg/dL | 444 [388 - 568] | 474 [415 - 542] |  | 488 [429 - 585] | 385 [358 - 437] |
| APTT; seg. | 28.95 [26.73 - 31.53] | 29.00 [27.60 - 30.20] |  | 30.1 [26.4 - 31.2] | 29.8 [28.1 - 31.1] |

# Supplementary Figures

## Figure 1. Representative flow cytometry plots for Th0, Th1, Th2, Th17 and Treg cell subset identification
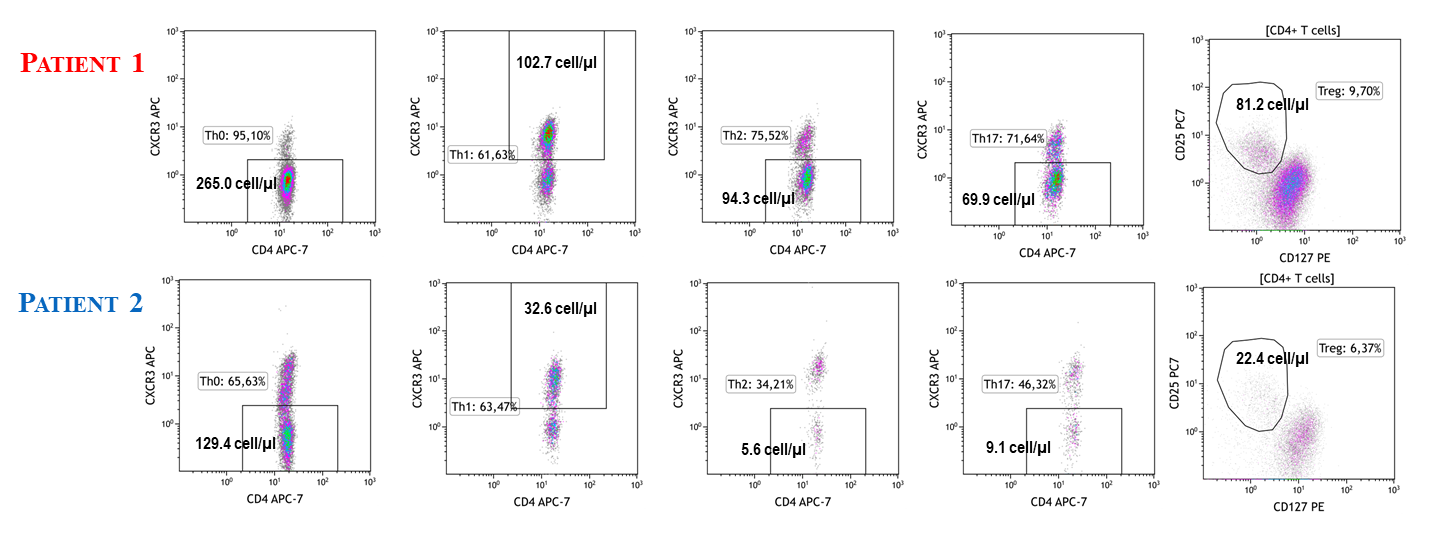


Antibody combinations: Th0: CD4+ CD45RA+ CCR6- CCR4- CXCR3-

Th1: CD4+ CD45RA- CCR6- CCR4- CXCR3+

Th2: CD4+ CD45RA- CCR6- CCR4+ CXCR3-

Th17: CD4+ CD45RA- CCR6+ CCR4+ CXCR3-

Treg: CD4+ CD25+ CD127low

## Figure 2. Variable contributions to cohort variability of the 6 main features of the dimensional reduction analysis for cardiovascular risk factors and medications.


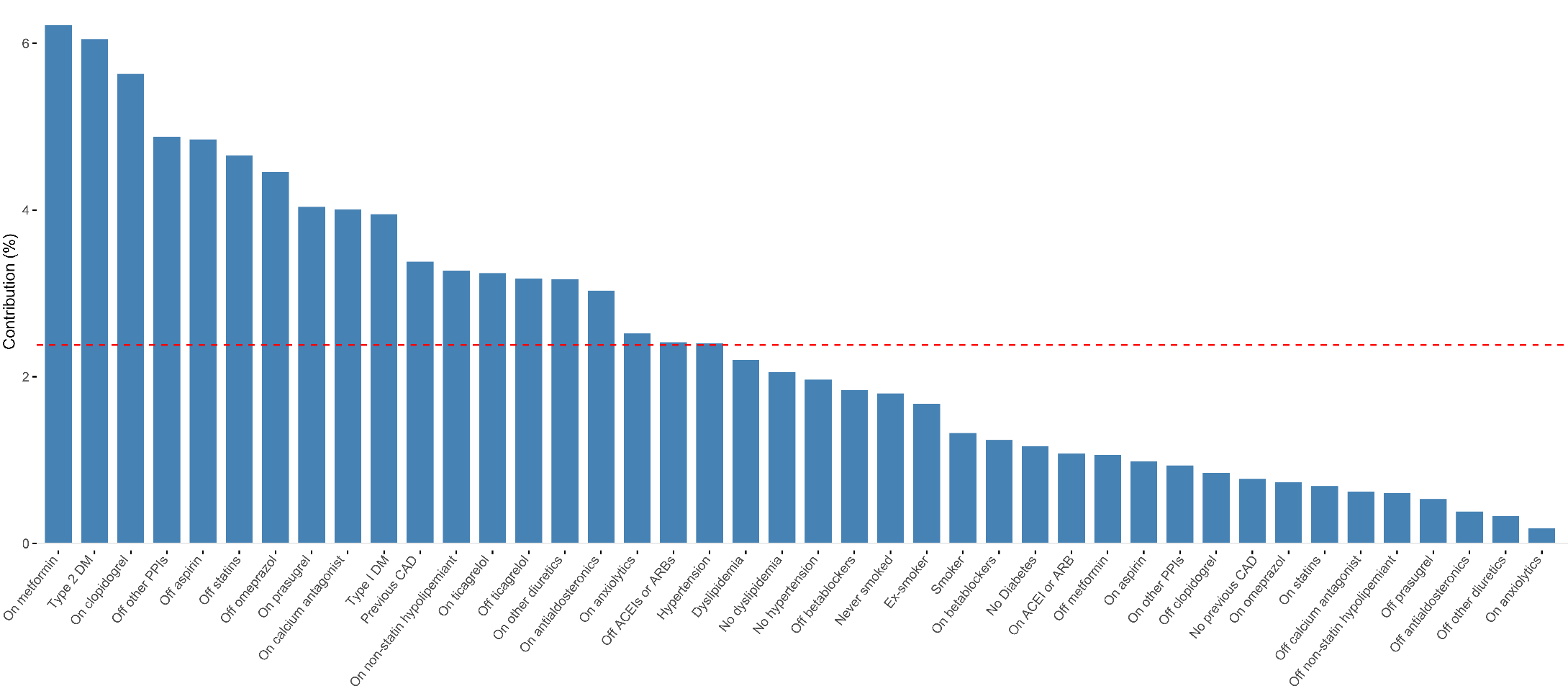


DM: Diabetes Mellitus; PPIs: Proton pump inhibitors; CAD: Coronary artery disease; ACEIs: Angiotensin-converting enzyme inhibitors; ARB: Angiotensin receptor blockers.

Off

## Figure 3. Adherence to the Mediterranean diet during follow-up assessed by 14-items MEDAS.


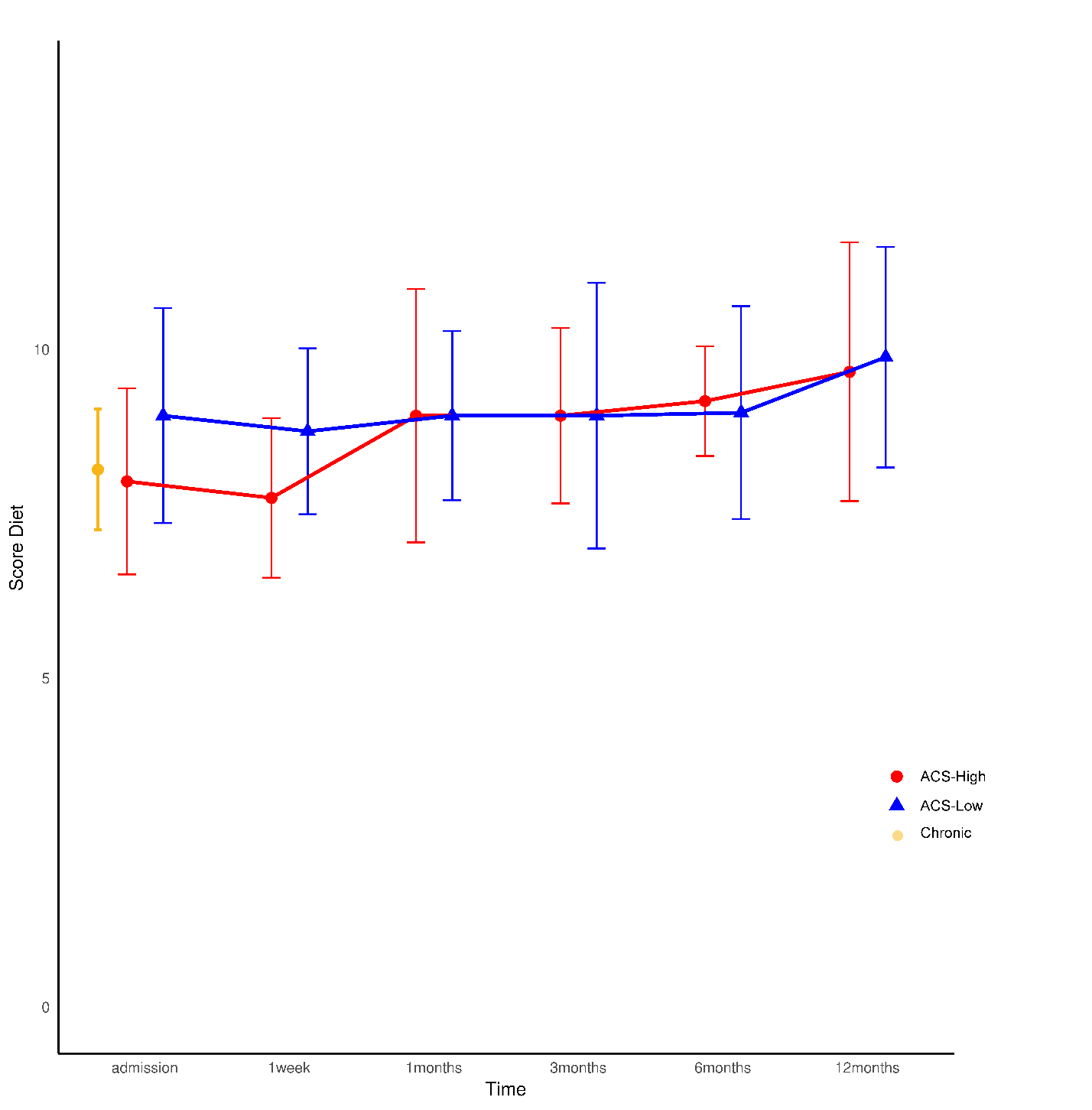


## Figure 4. Trends of the clustered cytokines significantly associated to high and low CD4+ T cell trajectories.


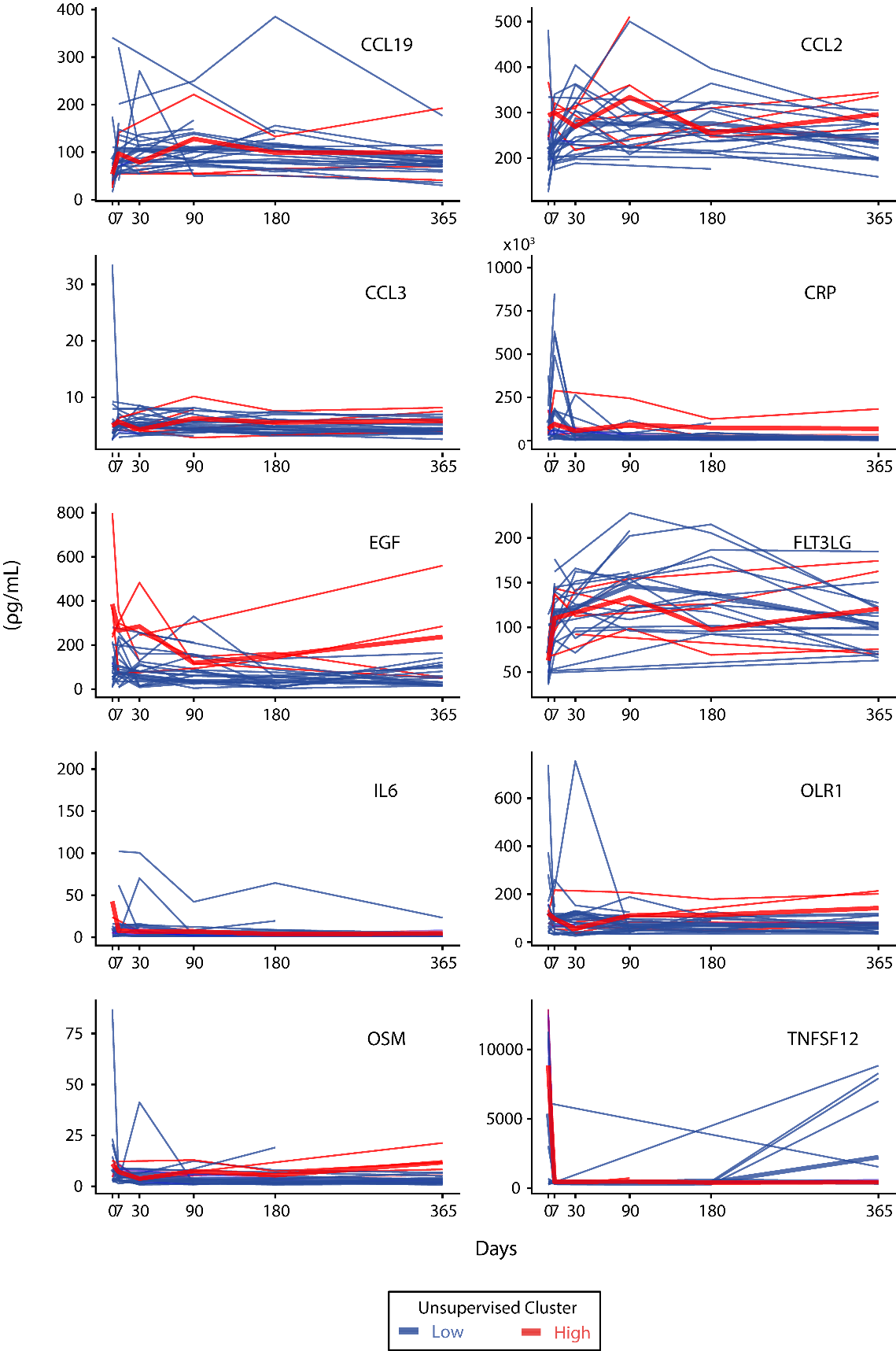


CCL9: C-C Motif Chemokine ligand 9; CCL2: C-C Motif Chemokine Ligand 2; CCL3: C-C Motif Chemokine Ligand 3; CRP: C-reactive protein; EGF: Epidermal growth factor, FLT3LG: Fms-related tyrosine kinase 3 ligand; IL6: Interleukin-6, OLR1: Ox-LDL receptor 1, OSM: Oncostatin M, TNFSF12: Tumor necrosis factor superfamily member 12.
